# Relationship between the experience of online game genre and high risk of Internet gaming disorder in Korean adolescents

**DOI:** 10.4178/epih.e2020016

**Published:** 2020-04-07

**Authors:** Hyunho Han, Hyunsuk Jeong, Sun-Jin Jo, Hye Jung Son, Hyeon Woo Yim

**Affiliations:** Department of Preventive Medicine, College of Medicine, The Catholic University of Korea, Seoul, Korea

**Keywords:** Adolescent, Online game, Internet gaming disorder, Game genre

## Abstract

**OBJECTIVES:**

This study examined the association between high risk of Internet gaming disorder (IGD) and online game genres used by adolescents.

**METHODS:**

The data derived from the baseline data of the Internet user Cohort for Unbiased Recognition of gaming disorder in Early Adolescence. A total 1,532 middle school students who use online games included. The participants reported the names of the online games they used during the past year. Game genres were categorized into role playing games (RPGs), shooting, multiplayer online battle arena (MOBA), simulation, arcade, sports and action games. The risk of IGD was measured using the Internet Gaming Use-Elicited symptom Screen. The relationship between the experience of online game genre and high risk of IGD was analyzed using multiple logistic regression model.

**RESULTS:**

The game time of a student was longer if he or she had an experience of RPGs, shooting games, MOBA games, simulation games, and action games. The direct and independent association between high risk of IGD in adolescents and the genres of RPGs, simulation games and MOBA were found to be odds ratios 1.52 (95% confidence interval [CI], 1.03 to 2.26); 1.59 (95% CI, 1.03 to 2.45); and 1.51 (95% CI, 1.03 to 2.21), respectively after adjusted the potential confounding variables and the use of other online game genres.

**CONCLUSIONS:**

The present cross-sectional study has found an association between online game genres and the risk of IGD in adolescents attending a school. A cohort study should verify the causal association in future.

## INTRODUCTION

Online games contain addictive factors that raise the immersion of the users, such as a gambling factor whereby the winning depends on luck, financial rewards through item dealings, and pursuit of a virtual reality that allows the formation of social relationships in the game space [1].

Previous studies reported that the risk of Internet gaming disorder (IGD) varies according to the game genre used by an individual. In a massively multiplayer online role-playing, the user sees the character in the game as oneself as it defeats enemies through upgraded attacks or defense abilities and excavated protective items such as weapons or shields, which increases the risk of gaming addiction [2]. Yee [3] reported that the factor driving the online role playing game (RPG) users towards gaming addiction was their immersion in the role they play in the game, through which they get a feeling of accomplishment when they win a match against someone else by creating increasingly stronger avatars throughout the game and feel satisfied with the social relationships formed in a virtual world [3]. Griffiths & Nuyens [4] defined the structural features of gaming addiction as sensory immersion, a feeling of accomplishment obtained from the game, and social interaction in a virtual world, and reported that the RPG genre had an association with gaming addiction.

On the other hand, previous studies regarding the influence of online game genre on the risk of IGD included those that surveyed the names of the most preferred online games by subject and then analyzed the corresponding game genres to evaluate the usage [5-9] as well as those that examined the most frequently used game genres [10-13]. In such cases, a reverse causation effect may be observed because an increase in the risk of addiction may raise the potential preference of the user towards certain games and the probability of using such games more frequently. Thus, when assessing the level of game exposure based on the genre of the most preferred game and that of the most frequently played game, the association between online game genres and the risk of IGD may be overestimated. An unbiased estimation of the pure association between online game genres and the risk of IGD may be possible by analyzing the exposure to a diversity of online game genres during the past year.

Most notably, new genres have been created through novelty or by combining existing genres [14]. For example, the genre of multiplayer online battle arena (MOBA) has been fully established with the creation of the game League of Legends in 2012. In addition, the game Minecraft can be seen as a combination of the RPG and simulation game genres [15,16]. Online games underwent flexible changes based on the preferences or consumer demands at each time, and the association between online game genres and the risk of IGD should be investigated afresh with each change in online game genres. Besides the combined online game genres, Korean adolescents were found to be using two or more online games simultaneously [17]. Such trends indicated a possibility that online game users are under a complex influence of multiple genres. Thus, the research should be adjusted to examine the association between online game genres and the risk of IGD, whether a subject is using multiple game genres at the same time.

The present study set out to investigate the effect of online game genres on the risk of IGD in adolescents attending school, and for this, the potential confounding variables and whether the subjects had been exposed to other online game genres were adjusted before examining the association between the exposure to an online game genre and the risk of IGD.

## MATERIALS AND METHODS

### Participants

The present investigation is a cross-sectional study analyzing the baseline data collected through the Internet user Cohort for Unbiased Recognition of gaming disorder in Early Adolescence (iCURE), a type of Internet user cohort [18]. The iCURE study recruited 2,319 students from 21 schools. Data were collected during class time using web-based self-reported. Of 2,319 students, 1,920 first-year middle school students within the cohort included to remove the age effect based on a previous study [7], IGD is affected by individual game expertise so that its level varies according to age with respect to the game genre preferred by adolescents. Of 1,920 students, 299 were excluded as they lacked the experience of playing online games in the past year; 32 were excluded as they either did not provide the name of the game or provided a name that could not be verified; 57 were excluded as they did not response on the question about daily spending time to play online game. A total 1,532 were analyzed in the study.

### Measurement tools

#### Online game genre

The subjects provided the names of the online game they experienced in the past year. As many as five online game names could be provided both personal computer games and mobile games. Based on the collected game names, the game use of subjects was classified according to genre. The game genre was categorized into RPGs, shooting, MOBA, simulation, arcade, sports, and action games, based on the content, the playing method (whether the game is played by a single player or multiple players and whether the game promotes collaboration or competition), the need of learning steps, and the type of reward system [14,17].

RPGs involve an avatar playing a specific role and growing in a game world that never ends; the most representative ones included Maple-Story and Dungeon Fighter. Shooting games involve a single-round game where the player wins by using a gun or a knife to annihilate the opponent. These games require quick reactions, and they are divided into first-person and third-person shooting games. The most representative one was Sudden Attack. Simulation games comprised the strategy simulation ones where the players compete through strategies to win a simulated war and the business/architecture/cultivation simulation ones where the players can have a virtual experience of an object or incident that exists or can exist in reality. The most representative ones included StarCraft and Minecraft. MOBA games are real-time action siege games based on strategy simulation methods. The most representative one was League of Legends. Arcade games are also called casual games, and they can be played in a relatively easy and simple way in terms of level of difficulty rather than progress methods. The most representative ones included Anipang and Sichuan. Sports games are based on a sport theme such as soccer, basketball and baseball. The most representative one was FIFA online. Action games included Lost Saga.

#### Risk of Internet gaming disorder

To measure the risk of IGD, the Internet Gaming Use-Elicited Symptom Screen (IGUESS) was used [19] which was a self-reported assessment developed by Diagnostic and Statistical Manual of Mental Disorders, 5th edition IGD criteria. This is a 4-point Likert scale from not at all (0) to always (3), with higher scores indicating a higher risk of IGD. The range of total scores was 0-27, and in this study, the subjects with 10 or more cut-off were categorized as having a high risk of IGD [19]. The confidence level of the tool was Cronbach’s α= 0.94 in a previous study, and the level was Cronbach’s α= 0.86 in this study.

#### Time spent on online gaming

Each subject provided the daily average time spent engaged in online gaming and the number of game playing days for weekdays and weekends in the past three months. Based on this, the total online game time during a week for a given subject could be estimated, and daily average online game time could be described. Next, in reference to a previous study where game users with ≥ 2 hours of daily average online game time were classified as a long-time user, the subjects in this study were divided into those with daily average 2 or more hours and those with daily average less than 2 hours [20].

#### Family type

The family type of each subject was categorized into non-intact family and intact family. If a subject lived with no parent, his or her family was categorized as non-intact. If a subject had one parent due to divorce or bereavement, his or her family was categorized as intact.

#### Socioeconomic status

The socioeconomic status was categorized into seven groups from 1 (lowest) to 7 (highest). The subjects in the range of 1-4 were categorized as having a low status and those in the range of 5-7 were categorized as having a high status.

#### Depressive symptoms

To measure the depressive symptoms, the Korean version of Children’s Depression Inventory (CDI) [21] was used. The original CDI was developed by Kovacs [22]. The CDI is a self-report measure that comprises 27 questionnaires regarding depressive feelings, social relationship problems, and helplessness, based on a 3-point Likert scale (0-point) and the range of total scores was 0-54 [23]. The confidence level in a previous study was Cronbach’s ɑ= 0.88, and it was Cronbach’s ɑ= 0.88 in this study.

#### Aggression levels

To measure aggression levels, the Korean version of the Aggression Questionnaire (AQ-K) [24] was used. The original AQ was developed by Buss and Perry. The AQ-K comprised a total of 27 questionnaires as a self-report measure for physical aggression, verbal aggression, anger and hostility as sub-elements. It is based on a 5-point Likert scale from extremely uncharacteristic (1) to extremely characteristic (5), with higher scores indicating higher aggression levels. The range of total scores was 27-135. The confidence level in a previous study was Cronbach’s ɑ= 0.86, and it was Cronbach’s ɑ= 0.89 in this study.

### Data analysis

For socio-demographic characteristics of subjects, the frequency and percentage were calculated for binary variables, and the mean and standard deviation were calculated for continuous variables.

The median and interquartile range were calculated for the daily average online game time based on whether the subject has experienced an online game genre in the past year, and for each online game genre, the Mann-Whitney U test was carried out to identify differences in the daily online game time. To understand the current state of the online game genres used by adolescents, the subjects were divided into those who have experienced a given online game genre and those who haven’t. Then based on the number of added genres, the frequency of subjects and the percentages out of the total number of subjects were estimated.

To investigate the differences in the risk of IGD depending on online game genres, a 3-step multiple logistic regression analysis was carried out. In model I, the odds ratio (OR) was calculated without adjusting the confounding variables. In statistics, 10-15 cases are required for the estimation of a variable in order to obtain unbiased values. Thus, around 10 confounding variables could be adjusted in this study as the outcome variable (high risk of IGD) had 154 prevalence cases (10.1%). In model II, the OR was calculated after adjusting the potential confounding variables such as gender, family type, economic status, depressive symptoms and aggression levels [25]. As adolescents use multiple game genres simultaneously, the online game genre usage was regarded as an additional confounding variable in model III to examine the direct and independent influence of each genre. Thus, a total of 11 confounding variables were adjusted so as to investigate whether the risk of IGD varies according to the characteristics of individual game genres.

All data were analyzed using the SAS version 9.4 (SAS Institute Inc., Cary, NC, USA), with the significance level set to ≤ 0.05. The type I error due to multivariate comparison was not corrected.

### Ethics statement

This study extracted and analyzed data from the iCURE cohort study and was approved by the Institutional Review Board of The Catholic University of Korea (No. MC19ZASI0130).

## RESULTS

The general characteristics of subjects showed that they included 1,036 boys (67.6%) and 496 girls (32.4%). The number of subjects with positive depressive symptoms was 86 (5.6%). The number of subjects with 2 or more hours of daily average online game time was 477 (31.1%). The number of subjects classified as having an intact family was 1,394 (91.0%) and those with a low or moderate socioeconomic status was 1,081 (70.6%). The game experience during the past year for each online game genre was as follows: RPGs, 608 students (39.7%); shooting games, 669 students (43.7%); MOBA, 599 students (39.1%); simulation games, 636 students (41.5%); arcade games, 1,001 students (65.3%); sports games, 371 students (24.2%); action game, 72 students (4.7%). The number of subjects showing a high risk of IGD was 154 (10.1%) (Table 1).

The daily average game time of the subjects with an experience of RPGs was 1.5 hours (0.7-2.7), which was significantly longer than 1.0 hour (0.4-2.0) of those without the experience (p<0.001). The daily average game time of the subjects with an experience of shooting games, MOBA games and action games was also significantly longer than that of those without the experience (p<0.001). No difference was found in the daily average game time between the subjects with an experience of arcade games and sports games and those without the experience (p=0.848 and p=0.539, respectively). Among the RPG users, only 15 (1.0%) was reported than thy RPGS only; those who use one more genre in addition to RPGs was 139 (9.1%); those who use two more additional genres was 188 (12.3%); those who use three more genres was 153 (10.0%); those who use four more genres was 113 (7.4%). In the case of shooting games, MOBA and action games, likewise, the subjects showed a tendency to use several different online game genres. Interestingly, the number of subjects who use arcade games only was 199 (13.0), while there was no subject who solely used action games (Table 2).

Analyzing the association between the online game genre usage in the past year and the risk of IGD showed that the crude ORs of RPG users for high risk of IGD was 1.69 (95% confidence interval [CI], 1.21 to 2.36), shooting game users was 1.58 (95% CI, 1.13 to 2.21) and simulation game users was 1.65 (95% CI, 1.17 to 2.29). In model II, the adjusted ORs of RPGs, simulation games, and MOBA users for high risk of IGD were 1.63 (95% CI, 1.11 to 2.41), 1.48 (95% CI, 1.02 to 2.15), and 1.69 (95% CI, 1.11 to 2.57), respectively after adjusting the confounding factors including gender, family type, socioeconomic status, depressive symptoms and aggression levels. In model III, the adjusted ORs of RPG, simulation game, and MOBA users for high risk of IGD were 1.52 (95% CI, 1.03 to 2.26), 1.51 (95% CI, 1.03 to 2.21), and 1.59 (95% CI, 1.03 to 2.45), respectively adjusting online game genre as well as gender, family type, socioeconomic status, depressive symptoms, aggression levels, and online game genre (Table 3).

## DISCUSSION

This study investigated the association between online game genre and the risk of IGD in adolescents attending school. The study subjects were online game users only and the general characteristics showed that the percentage of boys (67.7%) was higher than girls. In general, the rate of game use based on gender tends to be higher in men than in women. The 2015 survey of the rate of game use in the general public found that, across all online game users, the proportion of men was 66.3%, a trend in line with the present investigation [17]. Meanwhile, the prevalence of IGD in individuals with a high risk was 10.1% in this study, which was similar to 11.5% and 9.0% reported by other previous studies on the prevalence of IGD [26,27]. However, due to differences in the tools applied in each study, an absolute comparison of prevalence in individuals with a high risk could not be performed.

The previous studies showed that the game time was the longest in the group using a RPG, followed by that using a first-person shooting game and that using a real-time strategy simulation game [11,28]. The comparison among the groups using a specific game genre in previous studies reported the relationship between online game genre and game time. The present study compared the game time between the users and non-users of each game genre rather than among the groups using each genre, thereby providing a set of unprecedented data. Meanwhile, the results of this study indicated that for the following 5 game genres (RPG, shooting, MOBA, simulation, and action games), the subjects with an experience of each genre showed longer game time than those without the experience. The online game time is one of the main risk factors of IGD, and it is a factor for which studies have reported a correlation between longer game time and higher risk of IGD [29]. An increase in game time may be attributed to the various inherent characteristics of the game for the immersion of the user, and related factors include the deep social relationship, reward, and level of accomplishment within the game [10, 25].

As first-year middle school students were using several online game genres simultaneously, the experience of each game genre was adjusted as a covariate, and the resulting independent association was also found to be significant for RPG, simulation game, and MOBA. A previous study reported that the use of three game genres (RPG, action and adventure games), had an association with IGD [30], while MOBA was also reported as a risk factor showing an association with IGD in yet another study [31]. These results collectively point towards the potential risk of the use of RPGs and MOBA, as found significant in this study. However, the risk of the use of simulation games, as found significant along with RPGs and MOBA in this study, was not shown to exhibit a significant difference in other previous studies. Such deviation in results may be due to the differences in the categorization system for the simulation game genre, the recent incorporation of addictive factors in the simulation game genre, or the change in the environment as YouTube has extended the distribution of gaming videos.

Thus, considering the risk of the use of RPGs, MOBA, and simulation games, the association with high risk of IGD is thought to have increased due to the inherent characteristics of the game for each genre. The main characteristics of RPGs shows that they may offer an environment for the players to form social interactions easily [3,32,33]. Factors such as manipulation of personal avatars, a wide spectrum of virtual worlds, and the guild or clan systems, maintain deep and longstanding social interactions among players [3,32,33]. This enables the game users to relieve the negative feelings they might have through such social interactions and satisfy the social needs that had been insufficient in reality. As a result, the users are driven to use the online game for a longer period of time, which is likely to increase the potential risk of IGD. When it come to the difference between MOBA and RPG, MOBA also emphasizes teamwork solely for winning the game, compared to RPGs [31]. In other words, the social interaction in RPGs is for partnership, whereas that in MOBA is for teamwork. Hence, MOBA users would need to practice frequently so as not to interfere with the victory of the team, and the reward effect of victory is likely to increase the potential risk of IGD [31,34]. The main way a simulation game is played is for the user to use strategies to defeat an enemy in a war or to reproduce a specific circumstance on the computer [14]. Such gaming method necessitates more intellectual thinking than in other game genres, while inducing a competitive spirit in users, all of which raises the immersion of the users in the game. Unlike RPGs, simulation games have not been a genre reported as a main risk factor of IGD [11,28,33].

The simulation games in this study mostly comprised business/architecture/cultivation games that, along with the spread of broadcast games in 2015 [14], gained explosive popularity among adolescents in Korea. They represent a game genre that combines the characteristics of several genres such as cultivation, strategy, and adventure [15]. The business/architecture/cultivation simulation games can be played by adolescents relatively easily, while they construct an infinite variety of contents within the game [16]. Considering the characteristics of simulation games, the subjects in this study – the early adolescents who had just entered middle school, may have found it difficult to play these games in an immersive way as they require the learning of certain gaming abilities; for example, the entrance level of a first-person shooting game may have been too high for them [16]. On the contrary, as the business/architecture/cultivation simulation games can be used relatively easily by early adolescents as well [35], it is possible that these games raised an interest in the subjects, compared to other genres.

The limitations of this study are as follows. First, the study recruited the subjects from schools and thus excluded the adolescents not attending school, which prevents the generalization of the findings in this study to adolescents in all Korean communities. Second, although the subjects were recruited from schools, the characteristics of the schools such as the mood and policy regarding online games could not be taken into consideration for a multi level analysis. Third, we could not figure out how frequently they used each game genre could not be identified.

To conclude, the present cross-sectional study targeting adolescents attending school has found an association between online game genres and the risk of IGD. In future, a cohort study should be conducted to verify the causal association.

## Figures and Tables

**Table 1.** General characteristics of 1,532 first-year middle school students in Korea

Variables	n (%) or mean±SD
Gender	
Boys	1,036 (67.6)
Girls	496 (32.4)
Depressive symptoms	8.3±7.2
Aggression^1^	55.3±17.0
Average online-game time (hr/d)	
≥2	477 (31.1)
<2	1,055 (68.9)
Family type	
Non-intact family	138 (9.0)
Intact family	1,394 (91.0)
Social economic statue	
Low	1,081 (70.6)
High	451 (29.4)
Game genres^2^	
Role playing game	608 (39.7)
Shooting game	669 (43.7)
Multiplayer online battle arena	599 (39.1)
Simulation game	636 (41.5)
Arcade game	1,001 (65.3)
Sports game	371 (24.2)
Action game	72 (4.7)
Risk of Internet gaming disorder	
High^3^	154 (10.1)
Low^4^	1,378 (89.9)

1 Aggression score range: 27-135.

2 Multiple answer available.

3 High risk indicates 10 or higher scores in Internet Gaming Use-Elicited Symptom Screen.

4 Low risk indicates less than 10 in Internet Gaming Use-Elicited Symptom Screen.

**Table 2.** Experience of game genre, daily average time spent and number of game genres

Game genres^1^	Ever user during the past year	n	Average game time (hr/d)		No. of using game genres, n (%)^2^				
			Median (Q1-Q3)	p-value	1	2	3	4	5+
Role playing game	Yes	608	1.5 (0.7-2.7)	<0.001	15 (1.0)	139 (9.1)	188 (12.3)	153 (10.0)	113 (7.4)
	No	924	1.0 (0.4-2.0)		325 (21.2)	327 (21.3)	181 (11.8)	76 (5.0)	15 (1.0)
Shooting game	Yes	669	1.6 (0.7-2.7)	<0.001	19 (1.2)	151 (9.9)	199 (13.0)	177 (11.6)	123 (8.0)
	No	863	0.9 (0.3-1.9)		321 (21.0)	315 (20.6)	170 (11.1)	52 (3.4)	5 (0.3)
Multiple online battle arena	Yes	599	1.6 (0.7-2.6)	<0.001	31 (2.0)	108 (7.0)	187 (12.2)	162 (10.6)	111 (7.2)
	No	933	0.9 (0.3-2.0)		309 (20.2)	358 (23.4)	182 (11.9)	67 (4.4)	17 (1.1)
Simulation game	Yes	636	1.3 (0.5-2.5)	<0.001	43 (2.8)	189 (12.3)	181 (11.8)	126 (8.2)	97 (6.3)
	No	896	1.1 (0.4-2.2)		297 (19.4)	277 (18.1)	188 (12.3)	103 (6.7)	31 (2.0)
Arcade game	Yes	1,001	1.1 (0.4-2.3)	0.848	199 (13.0)	276 (18.1)	234 (15.3)	172 (11.2)	120 (7.8)
	No	531	1.2 (0.6-2.3)		141 (9.2)	190 (12.4)	135 (8.8)	57 (3.7)	8 (0.5)
Sports game	Yes	371	1.3 (0.5-2.3)	.539	33 (2.2)	58 (3.8)	105 (6.9)	104 (6.8)	71 (4.6)
	No	1,161	1.1 (0.5-2.3)		307 (20.0)	408 (26.6)	264 (17.2)	125 (8.2)	57 (3.7)
Action game	Yes	72	1.6 (0.8-3.1)	<0.001	0 (0.0)	11 (0.7)	13 (/0.8)	22 (1.4)	26 (1.7)
	No	1,460	1.1 (0.5-2.3)		340 (22.2)	455 (29.7)	356 (23.2)	207 (13.5)	102 (6.7)

1 Multiple answer available.

2 Numbers and proportions among 1,532 participants.

**Table 3.** Association between experience of game genre and risk of Internet gaming disorder (n=1,532)

Game genres	Category	Model I	Model II	Model III
Role playing game	Yes	1.69 (1.21, 2.36)	1.63 (1.11, 2.41)	1.52 (1.03, 2.26)
	No	1.00 (reference)	1.00 (reference)	1.00 (reference)
Shooting game	Yes	1.58 (1.13, 2.21)	1.39 (0.94, 2.06)	1.23 (0.82, 1.85)
	No	1.00 (reference)	1.00 (reference)	1.00 (reference)
Multiple online battle arena	Yes	1.38 (0.98, 1.92)	1.69 (1.11, 2.57)	1.59 (1.03, 2.45)
	No	1.00 (reference)	1.00 (reference)	1.00 (reference)
Simulation game	Yes	1.65 (1.17, 2.29)	1.48 (1.02, 2.15)	1.51 (1.03, 2.21)
	No	1.00 (reference)	1.00 (reference)	1.00 (reference)
Arcade game	Yes	1.08 (0.75, 1.53)	1.05 (0.69, 1.61)	1.01 (0.66, 1.53)
	No	1.00 (reference)	1.00 (reference)	1.00 (reference)
Sports game	Yes	0.99 (0.67, 1.45)	1.06 (0.68, 1.66)	1.13 (0.72, 1.78)
	No	1.00 (reference)	1.00 (reference)	1.00 (reference)
Action game	Yes	0.96 (0.43, 2.13)	0.57 (0.20, 1.64)	0.56 (0.19, 1.61)
	No	1.00 (reference)	1.00 (reference)	1.00 (reference)

Values are presented as odds ratio (95% confidence interval). Model II: adjusted by gender, family type, socioeconomic status, depressive symptom, and aggression; Model III: adjusted by gender, family type, socioeconomic status, depressive symptom, aggression, and online game genres.
