# Supplementary material for: Relationship between the experience of online game genre and high risk of Internet gaming disorder in Korean adolescents
Source: Epidemiol Health. 2020 Apr 7;42:e2020016. doi: 10.4178/epih.e2020016 (PMC7285446; doi:10.4178/epih.e2020016)
Supplement: Supplementary file 1 [file epih-42-e2020016-suppl.pdf]

Volume: 42, Article ID: e2020016
https://doi.org/10.4178/epih.e2020016

Relationship between online game genre used and high risk of Internet gaming disorder in adolescents

청소년에서 온라인 게임장르 이용경험과 인터넷게임장애 고위험과의 연관성

Original article

Hyunho Han*: https://orcid.org/0000-0002-5336-6351;
Hyunsuk Jeong*: https: //orcid.org/0000-0001-5274-3816;
Sun-Jin Jo*: https://orcid.org/0000-0002-8465-9632;
Hye Jung Son*: https://orcid.org/0000-0001-7256-0232;
Hyeon Woo Yim*: https://orcid.org/0000-0002-3646-8161;

*Department of Preventive Medicine, College of Medicine, The Catholic University of Korea, Seoul, Republic of Korea

This study was supported by a grant from the Korean Mental Health Technology R&D Project, Ministry of Health and Welfare, Republic of Korea (HL19C0012).

Corresponding author: Hyeon Woo Yim
Department of Preventive Medicine, College of Medicine, The Catholic University of Korea,
Address: 222, Banpo-daero, Seocho-gu, Seoul, Republic of Korea, 06591
Tel: +82-2-2258-7860 Fax: 02-532-3820 E-mail: y1693@catholic.ac.kr

The authors have no conflicts of interest to declare for this study.

Volume: 42, Article ID: e2020016
https://doi.org/10.4178/epih.e2020016

Volume: 42, Article ID: e2020016
https://doi.org/10.4178/epih.e2020016

ABSTRACT

Objectives: 온라인 게임에 몰입을 위해서 중독적 요소가 많이 삽입되는 것으로 알려져 있다. 청소년들이 이용하는 온라인 게임장르에 따른 인터넷게임장애 고위험과의 연관성을 알아보고자 하였다.
Methods: 서울 및 경기도지역에서 수행된 Internet user Cohort for Unbiased Recognition of gaming disorder in Early Adolescence (iCURE)의 기저시점 자료를 이용한 단면조사 연구이다. 온라인 게임을 이용하는 중학생 1,532명을 대상으로 분석하였다. 지난 1년간 이용했던 온라인 게임 이름들을 적은 후에 7개의 게임장르(롤플레잉게임, 슈팅게임, 멀티플레이어 온라인 배틀 아레나(MOBA), 시뮬레이션게임, 아케이드게임, 스포츠게임, 액션게임)로 구분하였다. 인터넷게임장애 위험은 Internet Gaming Use-Elicited symptom Screen(IGUESS)로 측정하였다. 온라인 게임장르 별로 사용 여부에 따른 하루평균 온라인 게임 이용시간을 비교하였다. 온라인 게임 장르별 사용여부에 따라 잠재적 혼란변수, 다른 온라인 게임 장르 사용여부를 보정한 교차비를 산출하였다.
Results: 롤플레잉게임, 슈팅게임, MOBA, 시뮬레이션게임, 액션게임 장르 이용 경험이 있는 경우 게임이용시간이 더 길었다. 잠재적 혼란변수와 다른 온라인 게임장르 사용여부를 보정한 상태에서 롤플레잉게임, 시뮬레이션게임, MOBA 장르는 청소년에서 인터넷게임장애 고위험에 대한 직접적, 독립적 연관성이 각각 OR=1.52 (95% CI: 1.03-2.26), OR=1.59 (95% CI: 1.03-2.45), OR=1.51 (95% CI: 1.03-2.21)로 나타났다.
Conclusions: 학교 기반 청소년들을 대상으로 한 단면조사연구에서 온라인 게임장르에 따른 인터넷게임장애 위험과의 연관성이 있었다. 코호트 연구를 통한 원인적 연관성 확인이 필요하다.

Key words: Adolescent, Online game, Internet gaming disorder, Game genre

Volume: 42, Article ID: e2020016
https://doi.org/10.4178/epih.e2020016

서론

온라인 게임에는 운에 따라 승패가 좌우되는 도박적 요소, 아이템 거래를 통한 금전적 보상, 게임 공간에서 대인관계를 맺을 수 있는 가상현실 지향 등과 같은 중독적인 요소가 있어서 온라인 게임에 몰입하도록 하는 역할을 하고 있다[1].

대상자가 이용하는 게임 장르에 따라 인터넷게임장애 위험에 차이가 있다고 보고되었다. 대규모 다중사용자 롤플레잉게임은 공격력이나 방어력을 업그레이드하고 무기나 방패 등 보호장비 아이템을 획득하여 적을 쳐부수는 게임 속 캐릭터를 분신처럼 여김으로 게임중독의 위험성을 높인다 [2]. Yee (2006)는 온라인 롤플레잉게임 이용자들은 게임을 통해 더 강한 아바타를 만들고 타인과의 대결에서 승리를 통해 성취감을 느끼며 가상세계에서 맺는 대인관계를 통해 만족감을 느끼며 게임에서 자신의 역할에 대한 몰입을 하게 되는 것이 게임중독으로 이끄는 요인이라고 보고하였다[3]. Griffith 등 (2017)은 게임중독의 구조적 특성을 감각적 몰입, 게임을 통해 얻은 성취감, 가상세계에서 타인과의 상호작용이라고 정의하면서 롤플레잉게임 장르가 게임중독과 연관되어 있음을 보고하였다 [4].

한편, 온라인 게임의 장르에 따른 인터넷게임장애 위험성에 미치는 영향을 알아본 연구에서 대상자가 가장 좋아하는 온라인 게임의 이름을 조사한 후, 해당 게임의 장르를 파악하여 이용여부를 평가한 연구들[5-9], 대상자가 가장 많이 이용하는 장르를 조사하여 평가한 연구들이 있었다[10-13]. 이 경우 중독위험성이 올라감에 따라 특정 게임을 더 좋아하게 될 가능성과 더 많이 이용할 가능성이 높아질 수 있으므로 역인과관계 (reverse causation) 효과가 있을 수 있다. 따라서 가장 좋아하는 게임의 장르와 가장 많이 사용하는 장르로 노출을 평가하는 경우 온라인 게임 장르에 따른 인터넷게임장애 위험성과의 연관성을 과대 추정할 가능성이 있다. 지난 1년간 다양한 온라인 게임 장르에 노출 여부를 파악하여 분석한다면 순수하게 온라인 게임 장르에 따른 인터넷게임장애 위험성과의 연관성을 편의 되지 않게 측정 가능할 것이다.

온라인 게임은 초창기라 할 수 있는 2000년대에 비해 온라인 게임은 많은 변화 과정을 거쳐왔으며, 특히 새로운 장르의 탄생 혹은 기존의 장르를 섞은 혼합된 장르가 등장해 왔다[14]. 예를 들어, 2012년 리그오브레전드 등장으로 멀티 플레이어 온라인 배틀 아레나(MOBA)는 확실한 게임 장르 중 하나로 자리매김하였다. 또한, 마인크래프트는 롤플레잉게임과 시뮬레이션 장르가 섞였다 할 수 있다[15,16]. 온라인 게임은 당시의 유행이나 소비자의 요구에 따라 유연하게 변화해왔으며, 온라인 게임장르 변화에 따라 온라인 게임장르와 인터넷게임장애 위험성과의 연관성을 새로이 조사해야 될 필요성이 있다. 혼합된 온라인 게임장르 등장과 더불어, 한국의 청소년들은 2개이상의 온라인 게임을 이용하는 것으로 나타났다[17]. 이러한 경향으로 인해 온라인 게임이용자들은 장르의 영향을 복합적으로 받고 있을 가능성이 있다. 따라서 온라인 게임장르와 인터넷게임장애 위험성과의 연관성을 살펴보기 위해서는 동시에 이용하고 있는 다른 게임장르사용여부를 보정하는 과정이 필요하다.

본연구에서는 학교 기반 청소년에서 온라인 게임 장르에 따른 인터넷게임장애 위험성에 미치는 효과를 알아보기 위하여 잠재적 혼란변수와 다른 온라인 게임장르 노출여부를 보정한 후 온라인 게임장르 노출 여부와 인터넷게임장애 위험성과의 연관성을 알아보고자 하였다.

Volume: 42, Article ID: e2020016
https://doi.org/10.4178/epih.e2020016

대상 및 방법

대상자

본 연구는 인터넷 사용자 코호트인 Internet user Cohort for Unbiased Recognition of gaming disorder in Early Adolescence (iCURE)[18] 에서 기저시점에 수집된 자료를 내려 받아 분석한 단면 연구이다. iCURE 연구는 2015년 5월부터 2017년 8월까지 서울, 경기도에 소재한 6개 초등학교와 15개 중학교 총 21개 학교에서 2,319명이 등록되었다. 조사는 훈련받은 연구원의 안내에 따라 수업시간에 컴퓨터 입력 방식으로 진행되었다. 참여자에게는 인증코드가 부여되었고, 각자의 인증코트 입력 후 자가보고로 이루어졌고 소요시간은 2시간이었다. 한편, 인터넷게임장애는 게임의 숙련도에 영향을 받기 때문에 청소년을 대상으로 선호하는 게임장르에 따른 인터넷게임장애의 정도가 연령별로 다르게 나타난 선행연구를 참고하여[7], 연령효과를 제거하기 위해 코호트 내 중학교 1학년생 1,920명을 대상으로 하였다. 연구목적을 위해 조사일 기준 지난 1년간 온라인 게임 이용경험이 없는 자 299명을 제외하였고, 기입한 게임의 이름 파악이 불가능하거나 미기입한 자 32명, 게임이용시간을 입력하지 않은 자 57명을 제외하였다. 이에 따라, 최종 1,532명이 분석되었다.

측정도구

온라인 게임장르

대상자들은 조사일 기준 지난 1 년간 이용했던 온라인 게임의 이름을 PC(personal computer)게임과 모바일게임으로 나누어 각각 5 개까지 기입하였고, 얻어진 게임 이름을 통해 장르별로 사용여부를 분류하였다. 게임장르의 구분은 게임의 내용과, 게임진행방식 (혼자서도 가능한 게임 혹은 여러 명이 참여해야 가능한 게임 여부, 협력 혹은 경쟁 게임 여부), 숙련과정이 필요한지 여부, 보상체계유형 등에 따라 롤플레잉게임, 슈팅게임, MOBA, 시뮬레이션게임, 아케이드게임, 스포츠게임, 액션게임으로 장르를 분류하였다[14,17]. 롤플레잉게임은 끝나지 않는 게임세계 속에서 아바타가 특정한 역할을 수행하며 성장해 나가는 방식으로, 대표적인 게임은 메이플스토리, 던전앤파이터 등이 있었다. 슈팅게임은 단판으로 진행되며 총, 칼 등을 사용해 상대편을 전멸시키면 승리하는 방식이다. 이는 빠른 순발력을 필요로 하며, 이용자의 시점에 따라 1 인칭슈팅게임과 3 인칭슈팅게임으로 구분된다. 대표적인 게임으로 서든어택 등이 있었다. 시뮬레이션게임은, 모의 전쟁에서 승리를 위해 서로의 전략으로 승부하는 전략시뮬레이션과, 실제로 존재하거나 존재할 수 있는 사물 및 사건을 가상으로 체험하는 경영/건설/육성 시뮬레이션을 포함시켰다. 대표적인 게임으로는 스타크래프트와 마인크래프트 등이 있었다. MOBA 는 실시간 액션 공성게임으로, 전략시뮬레이션 방식으로 진행되면서, 롤플레잉게임의 특징 또한 나타난다. 대표적인 게임으로 리그오브레전드가 있었다. 아케이드게임은 캐주얼 게임이라고도 하며, 진행방식이 아닌 난이도 측면에서 비교적 쉽고 간단하게 플레이 할

Volume: 42, Article ID: e2020016
https://doi.org/10.4178/epih.e2020016

수 있는 게임이다. 대표적으로 애니팡, 사천성 등이 있었다. 스포츠게임은 축구, 농구, 야구와 같은 스포츠를 소재로 진행되며, 대표적인 게임으로는 피파온라인이 있었다. 액션게임에는 로스트사가 등이 포함되었다.

인터넷게임장애 위험

인터넷게임장애 위험(Risk of Internet gaming disorder)은 Jo 등 (2018)이 개발한 Internet Gaming Use-Elicited Symptom Screen (IGUESS)척도가 활용되었다[19]. 이 척도는 DSM(Diagnostic and Statistical Manual of Mental Disorders)-5의 인터넷게임장애(Internet gaming disorder) 진단기준에 근거한 9개의 문항으로 구성되어 있는 자가보고형 도구로서, 아동기 이상의 연령에 대한 인터넷게임장애 증상을 선별한다. '전혀 아님(0점)'부터 '항상(3점)'까지 4점 리커트 척도로 되어있으며, 점수가 높을수록 인터넷게임장애 위험 수준이 높은 것으로 해석된다. 총점의 범위는 0-27점이며 본 연구에서는 절단점 10점 이상의 대상자들을 인터넷게임장애 고위험자로 구분하였다[19]. 선행연구에서 도구의 신뢰도는 Cronbach's α=.94로 나타났으며, 본 연구에서는 Cronbach's α=.86로 나타났다.

온라인 게임 이용시간

대상자들은 조사일 기준, 지난 3개월 동안 본인의 하루 평균 온라인 게임 이용시간과 이용 날수를 주중과 주말로 나누어 기입하였다. 이를 통해 대상자가 일주일 동안 온라인 게임을 이용하는 총 시간을 산출할 수 있었고, 하루 평균 온라인 게임 이용시간으로 나타낼 수 있었다. 그 후, 하루평균 온라인 게임을 2시간 이상 이용할 때 장시간 이용자로 구분한 선행연구를 참고하여, 하루 평균 2시간 이상 이용하는 대상자와 2시간 미만 이용하는 대상자로 구분하였다[20].

가족 유형

대상자들의 가족유형을 결손가정과 비결손가정으로 구분하였다. 부, 모가 모두 없는 대상자를 결손가정으로 분류했으며, 이혼, 사별 등의 이유로 부 또는 모 둘 중 한명이라도 함께 살고 있다면 비결손가정으로 분류하였다.

사회 경제적 지위

사회경제적 지위는 7단계로 구분하였으며, 1점(가장 낮음)부터 7점(가장 높음)으로 나타내었다. 1-4점에 해당하는 대상자를 '낮음', 5-7점에 해당하는 대상자를 '높음'으로 분류하였다.

우울증상

7-17세의 소아 청소년의 우울증상을 측정하기 위해 Kovac[21]이 개발한 Children's Depression Inventory(CDI)척도를 한국판으로 번안한 한국형 소아우울척도[22]를 활용하였다. 이 척도는 27문항으로 구성되어 있는 자가보고형 도구로서 우울한 기분, 대인관계 문제, 무력감 등에 대해 질문하는 내용으로 구성되어 있다. 0-2점까지 3점 리커트 척도로 되어있으며, 총점의 범위는 0-54점이다 [23]. 선행연구에서 도구의 신뢰도는 Cronbach's α=.88로 나타났으며, 본 연구에서는 Cronbach's α=.88로 나타났다.

공격성향

공격성향은 Buss&Perry가 공격성 측정을 위해 제작한 척도를 번안한 한국어판 공격성 척도(Korean version of the Aggression Questionnaire, AQ-K)[24] 를 활용하였다. 이 척도는 총 27문항으로 구성되어있으며, 신체적 공격성, 언어적 공격성, 분노감, 적대감을 하위영역으로 갖는 자가보고형 척도이다. '전혀 그렇지 않다(1점)'부터 '매우 그렇다(5점)'까지 5점 리커트 척도로 되어있으며, 점수가 높을수록 공격성향이 높은 것을 나타낸다. 총점의 범위는 27-135점이다. 선행연구에서 도구의 신뢰도는 Cronbach's α=.86로 나타났으며, 본 연구에서는 Cronbach's α=.89로 나타났다.

자료분석

대상자의 인구사회학적 특징에 대하여 이분형 변수의 경우 빈도와 백분율을, 연속형 변수의 경우 평균과 표준편차를 산출하였다.

지난 1년간 온라인 게임 장르 별로 이용 경험 유무에 따른 하루평균 온라인 게임 이용시간의 중앙값과 사분위수 범위를 산출하였고, 온라인 게임 장르 별로 하루 온라인 게임 이용시간

에 차이가 있는지를 알아보기 위해 Mann-Whitney U test를 실시하였다. 청소년들이 이용하는 온라인 게임 장르의 이용실태를 파악하기 위해 온라인 게임 장르별로 이용 경험 유무를 나눈 후에 추가되는 장르의 개수에 따라 대상자 빈도와 전체 대상자에서의 백분율을 나타내었다.

온라인 게임 장르에 따른 인터넷게임장애 위험의 차이를 알아보기 위하여 3단계 다중로지스틱 회귀분석(multiple logistic regression analysis)을 실시하였다. Model I 에서는 혼란변수를 보정하지 않은 상태에서 교차비를 산출하였다. 통계학적으로 편의되지 않는 값을 추정하기 위해서는 1개의 변수 추정에 10-15건의 사건이 필요하다. 이에 본 연구에서는 결과변수(인터넷게임장애 고위험)의 유병건수가 154명(10.1%)이기 때문에 대략 10개의 혼란변수를 보정할 수 있다. Model II 에서는 성별, 가족유형, 경제수준, 우울증상, 공격성향과 같은 잠재적 혼란변수를 보정하여 교차비를 산출하였다[25]. 청소년들이 다수의 게임장르를 동시에 이용하고 있으므로 장르의 직접, 독립적인 효과를 알아보기 위하여 Model III에서는 혼란변수로 다른 온라인 게임 장르 사용여부를 추가하였다. 따라서, 총 11개의 혼란변수를 보정하여 게임장르가 가진 특성에 따라 인터넷게임장애 위험성이 다른 지를 알아보고자 하였다.

모든 자료는 SAS 버전 9.4 통계패키지 프로그램을 이용하여 분석되었고, 유의수준은 0.05이하로 설정하였다. 다중비교에 의한 type 1 error는 보정하지 않았다.

결과

대상자의 일반적 특성으로 남학생이 1,036명(67.6%), 여학생이 496명(32.4%)이었다. 우울증상 양성은 86명(5.6%) 이었다. 하루 평균 온라인 게임 이용시간이 2시간 이상인 대상자는 477명(31.1%)이었으며 결손가정인 대상자는 1,394명(90.9%), 사회경제적 지위가 낮은 대상자가 1,081명(70.5%)로 나타났다. 지난 1년간 게임을 이용했던 경험은 온라인 게임 장르별로, 롤플레잉게임 608명(39.7%), 슈팅게임 669명(43.7), MOBA 599명(39.1%), 시뮬레이션게임 636명(41.5%), 아케이드게임 1,001명(65.3%), 스포츠게임 371명(24.2%), 액션게임 72명(4.7%)이었다. 인터넷게임장애 고위험자는 154명(10.1%)이었다. (Table1)

롤플레잉게임 이용경험이 있는 경우는 하루평균이용시간이 1.5(0.7-2.7)시간으로, 이용 경험이 없는 경우의 1.0(0.4-2.0)시간에 비하여 유의하게 길었다($p<.001$). 슈팅게임, MOBA, 액션게임의 이용경험이 있는 경우에도 해당 장르 경험이 없는 경우에 비해 유의하게 하루 평균 온라인 게임 이용시간이 길었다($p<.001$). 아케이드게임과 스포츠게임이용경험 유무에 따른 하루 평균 온라인 게임 이용시간의 차이는 나타나지 않았다($p=.848$, $p=.539$ 각각). 롤플레잉게임의 경우 롤플레잉게임만 이용하는 대상자는 15명(0.9%), 롤플레잉게임 외 1개 장르를 더 이용하는 경우 139명(9.1%), 2개 장르를 더 이용하는 경우 188명(12.3%), 3개 장르 더 이용하는 경우 153명(9.9%), 4개 이상 장르를 더 이용하는 경우 113명(7.4%) 이었다. 슈팅게임, MOBA, 액션게임으로 구분하였을 때도 여러 개의 온라인 게임 장르를 이용하는 분포를 보였다. 흥미롭게도 아케이드게임 하나 만을 이용하는 경우는 199명(12.9%)이 있었고, 액션게임 하나만을 이용하는 경우는 없었다. (Table2)

지난 1년간 온라인 게임 장르 이용경험과 인터넷게임장애 위험과의 연관성 분석에서 롤플레잉게임 이용경험자는 온라인 게임장애 고위험에 대한 혼란효과를 교정하지 않은 교차비(Odds Ratio, OR)가 1.69 (95% CI: 1.21-2.36) 이었고, 슈팅게임 장르 이용경험자는 인터넷게임장애 고위험에 대한 교차비가 1.58 (95% CI: 1.13-2.21) 이었다. 시뮬레이션게임은 이용경험자는 인터넷게임장애 고위험에 대한 교차비가 1.65 (95% CI: 1.17-2.29)이었다. 성별, 가족유형, 사회경제적 수준, 우울증상, 공격성향을 보정한 후에 롤플레잉게임, 시뮬레이션게임, MOBA이용경험자와 인터넷게임장애 고위험에 대한 교차비가 유의하게 높았다. 각각의 교차비는 1.64 (95% CI: 1.14-2.37), 1.71 (95% CI: 1.20-2.43), 1.48 (95% CI: 1.02-2.15) 이었다. 온라인 게임장르를 추가로 보정한 모델 III에서도 롤플레잉게임, 시뮬레이션게임, MOBA이용경험자의 인터넷게임장애 고위험에 대한 교차비가 유의하게 높았다. 각각의 교차비는 1.47 (95% CI: 1.01-2.14), 1.65 (95% CI: 1.15-2.38), 1.51 (95% CI: 1.03-2.21)로 나타났다. (Table3)

고찰

본 연구는 온라인 게임장르에 따른 인터넷게임장애 위험과의 연관성을 학교 기반 청소년을 대상으로 알아보고자 하였다. 온라인 게임 경험자만을 대상으로 한 본 연구대

상자의 일반적인 특성에서 남학생이 전체의 67.6%로 여학생보다 더 많은 비율을 차지하고 있었다. 일반적으로 성별에 따른 게임 이용률은 여성보다 남성에서 더 높게 나타나는 경향이 있다. 2015년 일반 국민의 게임 이용률 조사결과, 전체 온라인 게임 이용자 중 남성이 차지하는 비율이 66.3% 로 나타나, 본 연구와 유사한 경향을 확인할 수 있었다[17]. 한편, 본 연구에서 인터넷게임장애 고위험자의 유병률은 10.1%로 나타났다. 이러한 결과는 인터넷게임장애 유병률을 살펴본 다른 선행연구들의 결과인 11.5%, 9.0%의 유병률과 유사한 것을 확인할 수 있었다[26-27]. 하지만, 각 연구에서 사용한 도구의 차이로 인해 인터넷게임장애 고위험자 유병률의 절대적인 비교는 불가능 하였다.

온라인 게임장르와 게임이용시간에 대한 선행연구들의 결과를 살펴보면, 롤플레잉게임, 1인칭 슈팅게임, 실시간 전략시뮬레이션게임을 이용하는 그룹 순으로 게임이용시간이 길다고 보고되어 왔다[11,28]. 즉, 선행연구 에서는 특정 장르를 이용하는 집단 간의 비교를 통해 온라인 게임장르와 게임이용시간의 관계를 설명했다고 할 수 있다. 이에, 본 연구에서는 장르이용 집단 간의 비교가 아닌, 각 장르 이용자와 이용하지 않는 자의 게임이용시간을 비교하여 기존에 보고되지 않은 정보를 제시하였다고 볼 수 있다. 한편, 본 연구의 결과로 롤플레잉게임, 슈팅게임, MOBA, 시뮬레이션게임, 액션게임 5개의 장르에서 각 장르의 이용경험이 있는 대상자들이 없는 대상자보다 게임이용시간이 더 긴 것으로 나타났다. 온라인 게임 이용시간은 인터넷게임장애의 주요한 위험요인 중 하나로, 게임이용시간이 길수록 인터넷게임장애에 빠질 가능성이 높아지는 것으로 보고되어 온 요인이다[29]. 게임이용시간의 증가는 게임에 몰입을 시키기 위한 게임에 내제된 여러 특성 때문일 수 있으며 게임 내 깊은 사회적 관계, 보상, 성취의 정도 등이 요인이라 할 수 있다[10,25].

성별, 가족유형, 사회경제적 수준, 우울증상, 공격성향의 혼란효과를 보정한 상태에서 롤플레잉게임과 시뮬레이션게임, MOBA의 이용경험이 인터넷게임장애 고위험의 연관성을 유의하게 높였으며, 중학교 1학년 청소년들이 여러 개의 온라인 게임장르를 한꺼번에 사용하고 있었기 때문에 개별 장르의 사용여부를 보정하여 얻는 독립적인 연관성 또한 롤플레잉게임, 시뮬레이션게임, MOBA의 이용경험에서 유의한 연관성을 얻었다. 본 연구와 유사한 선행연구를 살펴보면, 롤플레잉게임, 실시간 전략시뮬레이션게임, 스포츠게임, 1인칭 슈팅게임 순으로 인터넷중독 위험자의 수가 많았다[11]. 또한, 다른 선행연구에서는 롤플레잉게임, 액션게임, 어드벤쳐게임 세 장르의 이용이 인터넷게임장애와 연관성을 보였으며[30], MOBA 또한 인터넷게임장애의 위험요인으로 관련성을 확인한 선행연구가 있었다[31]. 이러한 결과들은 본연구에서 유의하게 나타난 롤플레잉게임과 MOBA의 이용의 위험 가능성을 공통적으로 보여준다고 할 수 있다. 하지만, 롤플레잉게임, MOBA와 함께 유의하게 나타난 시뮬레이션게임 이용자의 위험성은 다른 선행연구에서는 나타나지 않는 차이를 보였다. 이러한 결과의 차이는 연구 별 시뮬레이션게임의 장르 분류체계의 차이, 최근 시뮬레이션게임 장르에 중독 요소의 가미, 혹은 유튜브를 통한 게임동영상의 확대로 인한 환경의 변화를 고려해 볼 수 있을 것이다. 이에, 본 연구에서 나타난 롤플레잉게임, MOBA, 시뮬레이션게임이용의 위험성에 대해 고려해 봤을 때, 각 게임장르에 내제된 게임의 특성으로 인해 인터넷게임장애 고위험과의 연관성이 높아진 것으로 사료된다. 롤플레잉게임에 대하여 살펴보면, 주요한 특징으로 사회적 상호작용(Social interaction)을 형성하는데 용이한 환경을 갖고 있다 할 수 있다[3,32-33]. 즉, 롤플레잉게임에서의 개인 아바타의 조작, 광범위한 가상세계, 길드/클랜 시스템 등이 이

용자들의 게임 내 사회적 상호작용(Social interaction)을 깊고 길게 유지시킨다[3,32-33]. 이에, 게임 이용자들은 사회적 상호작용(Social interaction)을 통해 부정적인 감정을 해소하고, 현실에서 부족했던 사회적 욕구를 충족하게 된다. 결국, 이용자들은 더 오랜 시간 온라인 게임을 이용하게 되고, 인터넷게임장애로 이어질 가능성이 높아지게 될 것이다. 또한, MOBA의 경우 약 30분~50분정도의 시간 내에 게임의 승패가 정해지는 것과 낯선 유저와 팀을 이루어 게임을 진행하는 경우가 비교적 많다는 것이 롤플레잉게임과의 큰 차이라 할 수 있다. 또한 롤플레잉게임에 비해 오로지 게임에서 승리하기 위한 팀워크가 더욱 중요시된다고 할 수 있다[31]. 즉, 롤플레잉게임은 친목성의 사회적 상호작용(Social interaction)이라면, MOBA는 팀워크를 위한 사회적 상호작용이라고 구분할 수 있을 것이다. 따라서, MOBA 이용자들은 팀의 승리를 위해 팀에 방해되지 않도록 많은 연습이 필요할 것이고, 승리를 통한 보상효과로 인터넷게임장애의 가능성이 높아질 수 있다[31,34]. 시뮬레이션게임은 전략을 통해 상대방을 전쟁에서 패배시키거나, 특정상황을 컴퓨터에서 재현하며 운영하는 것이 주된 방식이라 할 수 있다[14]. 이러한 진행 방식은 다른 장르의 게임에 비해서 이용자에게 지적인 능력을 요구하고, 경쟁의식을 발생시키는 특징을 갖게 하며, 이용자를 게임에 더욱 몰입하게 한다. 시뮬레이션게임은 롤플레잉게임과 비교해 봤을 때, 인터넷게임장애의 주요 위험요인으로 보고되어 온 장르는 아니었다[11,28,33]. 본 연구에서 시뮬레이션게임은 대부분 건설/경영/육성 시뮬레이션게임으로 구성되어 있다. 건설/경영/육성 시뮬레이션게임은 2015년도 '보는 게임'의 확산[14]과 함께 한국 청소년들 사이에서 폭발적으로 유행한 게임으로 육성, 전략, 모험과 같은 여러 장르의 특징이 복합적으로 적용된 대표적인 게임이라고 할 수 있다[15]. 건설/경영/육성 시뮬레이션게임은 청소년들이 비교적 쉽게 이용 가능하며, 게임 내 무궁무진한 컨텐츠를 제작할 수 있다[16]. 이와 같은 시뮬레이션게임의 특징을 고려해 봤을 때, 본 연구의 대상자는 중학교에 갓 진학한 어린 청소년들로, 1인칭 슈팅게임 등 게임실력을 익혀야 되는 진입장벽이 높은 게임들을 몰입적으로 이용하기 어려웠을 가능성이 있다[16]. 이에 반해, 건설/경영/육성 시뮬레이션게임은 어린 청소년도 비교적 쉽게 시작할 수 있기 때문에[35], 다른 게임에 비해 대상자의 흥미를 끌었을 가능성이 있다. 더욱이, 건설/경영/육성 시뮬레이션게임은 보는 게임 즉, 1인 미디어방송을 시청하며 플레이 방식을 따라 하며 배우기 좋은 특징을 갖고 있기 때문에[16], 1인 미디어방송의 시청효과로 대상자들은 해당 건설/경영/육성 시뮬레이션게임에 더욱 몰입했을 가능성도 있을 것이다. 본 연구에서 나타난 시뮬레이션게임과 인터넷게임장애 고위험과의 연관성은, 연구대상자의 연령수준에 따른 게임이용 능력과, 보는 게임의 확산이라는 연구 시점의 상황으로 인해 그 효과가 더욱 부각되었을 가능성이 있을 것으로 생각된다. 따라서, 추후 청소년, 특히 어린 청소년들의 온라인 게임장르 이용에 관한 연구 시, 대상자들의 연령수준에 따른 게임이용의 특징에 주목할 필요가 있으며, 보는 게임의 유행과 같은 해당 시점에 유행적인 요소의 파악이 중요할 수 있다.

본 연구의 제한점은, 첫째, 학교기반으로 대상자를 모집하였기 때문에 학교 밖 청소년이 배제되어 있고, 한국의 2개 도시지역에서 대상자를 모집하였기 때문에 한국의 지역사회 청소년을 대변한다고 보기 어렵다. 또한, 인터넷게임장애는 인터넷 인프라, 지역환경 및 문화 등의 영향을 받기 때문에 본 결과를 다른 나라의 청소년에 적용할 때는 주의가 필요하다. 둘째, 학교단위로 학생들이 등록되었음에서 불구하고 온게임에 대한 학교 내 분위기, 정책 등 학교 특성을 고려한 다수준 분석을 고려하지 못한 제한점이 있다.

https://doi.org/10.4178/epih.e2020016

셋째, 지난 1년간 온라인 게임장르 사용여부를 자가보고에 의해 게임명을 기술하게 하였기 때문에 해당 게임을 얼마나 많이 사용하는지에 대한 정량적인 정보는 알 수가 없었다.

결론적으로 학교 기반 청소년들 대상 단면조사연구에서 온라인 게임장르에 따른 인터넷게임장에 위험에 연관성이 있었다. 코호트 연구를 통한 원인적 연관성 확인이 필요하다.

Ethics statements

This study extracted and analyzed data from the iCURE cohort study and was approved by the institutional review board of the Catholic University of Korea (no. MC19ZASI0130).

ORCID

Hyunho Han: https://orcid.org/0000-0002-5336-6351; Hyeon Woo Yim: https://orcid.org/0000-0002-3646-8161; Hyunsuk Jeong: https: //orcid.org/0000-0001-5274-3816; Sun-Jin Jo: https://orcid.org/0000-0002-8465-9632; Hye Jung Son: https://orcid.org/0000-0001-7256-0232;

Volume: 42, Article ID: e2020016
https://doi.org/10.4178/epih.e2020016

REFERENCES

1. Kuss DJ. Internet gaming addiction: current perspectives. Psychol Res Behav Manag 2013;6:125-137.
2. Kimberly, Young. Addiction to MMORPG: Symptoms and Treatment. 2005 [cited 2020 Feb 21]. Available from: http://www.netaddiction.com/articles/addiction_to_mmorpgs.pdf
3. Yee N. The psychology of massively multi-user online role-playing games: Motivations, emotional investment, relationships and problematic usage. Avatars at work and play 2006;187-207.
4. Griffiths, M. Nuyens F. An Overview of Structural Characteristics in Problematic Video Game Playing. Current Addiction Reports 2017;4:272–283.
5. MS Lee, YH KO, HS SONG, KH KWON, HS LEE, M NAM et al. Characteristics of Internet use in relation to game genre in Korean adolescents. CyberPsychology & Behavior 2006;10:278-285.
6. T. Thorne, J. Smith, J. Morgan, J. Babic, R. Lubans. Video game genre preference, physical activity and screen-time in adolescent boys from low-income communities. Journal of adolescence 2014;37:1345-1352.
7. Adam E, Florian K, Daniel B, Douglas A, Choo HK, Vivian H. et al. The Role of Game Genres and the Development of Internet Gaming Disorder in School-Aged Children. J Addict Behav Ther Rehabil 2015;4
8. Beate Braun, Juliane M. Stopfer, Kai W. Müller, Manfred E. Beutel, Boris Egloff. Personality and video gaming: Comparing regular gamers, non-gamers, and gaming addicts and differentiating between game genres. Computers in Human Behavior 2016;55:406-412.
9. Stefano Tribertia, Luca Milanic, Daniela Villania, Serena Grumic, Sara Peracchiad, Giuseppe Curcioe, Giuseppe Riva. What matters is when you play: Investigating the relationship between online video games addiction and time spent playing over specific day phases. Addictive Behaviors Reports 2018;8:185-188.
10. You S, Jung E. A study on the influences of games in youth focusing on the heavy users. Studies on Korean Youth. 2001;12:35-63.
11. Kim JW, Han DH, Park DB, Min KJ, Na C, Won SK et al. The relationships between online game player biogenetic traits, playing time, and the genre of the game being played. Psychiatry Investig 2010;1:17.-23
12. Park BK, Shin SM, Lee HJ. A study on the relationship between types of teen preferred internet game and internet game addiction, parent-adolescent communication, and self-control. Korean J Health Psychol 2012;17:659-676.
13. Subramaniam, Chua, Abdin, Pang, Satghare, Vaingankar et al. Prevalence and correlates of Internet gaming problem among Internet users: Results from an Internet survey. Annals of the Academy of Medicine 2016;45:174-183.
14. Korea Creative Content Agency. 2016 White Paper on Korean Games; 2016[cited

2016 Nov 7]. Available from: http://www.kocca.kr/cop/bbs/view/B0000146/1831668.do?menuNo=201826.
15. Duncan SC. Minecraft, beyond construction and survival. a journal on video games 2011;1:1-22.
16. Karsenti PT, Bugmann J, Gros P. Transforming education with Minecraft. Results of an exploratory study 2017;118.
17. Ministry of Science and ICT. Survey on the internet usage; 2017[cited 2017 Mar 15]. Available from: https://isis.kisa.or.kr/board/index.jsp?pageId=060100&bbsId=7&itemId=817&search Key=&search Txt=&pageIndex=1.
18. Jeong H, Yim HW, Jo SJ, Lee SY, Kim E, Son HJ, et al. Study protocol of the Internet user Cohort for Unbiased Recognition of gaming disorder in Early adolescence (iCURE), Korea, 2015-2019. BMJ Open 2017;7:e018350.
19. Jo SJ, Yim HW, Lee HK, Lee HC, Choi JS, Baek KY. The Internet Game Use-Elicited Symptom Screen proved to be a valid tool for adolescents aged 10-19 years. Acta Paediatr 2018;107:511-516.
20. Ryu, Seung-ho, and Eui-jun Jeong. A study on the influences of games in youth-focusing on the heavy users. Studies on Korean Youth 2001;12:35-64.
21. Kovacs, M. CDI Children's Depression Inventory. Multi-Health Systems, New York 1992.
22. Cho SC, Lee YS. Development of the Korean form of the Kovacs' children's depression inventory. J Korean Neuropsychiatr Assoc 1990;11:943-956.
23. Yang JW, Kim YJ, Kim HS, Shin KM, Shin YM. Difference between children's self-reports on depression and parents assessment of children's behaviors. J Korean Acad Child Adolesc Psychiatry 2012;23:76-81.
24. Seo S, Kwon S. Validation study of the Korean version of the aggression questionnaire. Korean Journal of Clinical Psychology 2002;21:487-501.
25. Paulus FW, Ohmann S, von Gontard A, Popow C. Internet gaming disorder in children and adolescents: a systematic review. Dev Med Child Neurol. 2018;60:645-659.
26. Grusser SM, Thalemann R, Griffiths MD. Excessive computer game playing: evidence for addiction and aggression?. Cyberpsychology & behavior 2006;10:290-292.
27. Gentile DA, Choo H, Liau A, Sim T, Li D et al. Pathological video game use among youths: a two-year longitudinal study. Pediatrics 2011;127:e319-e329.
28. Ghuman D, Griffiths MD. A cross-genre study of online gaming: Player demographics, motivation for play, and social interactions among players. International Journal of Cyber Behavior, Psychology and Learning (IJCBPL) 2012;1:13-29.
29. Lemmens JS, Hendriks SJ. Addictive online games: Examining the relationship between game genres and Internet gaming disorder. Cyberpsychol Behav Soc

Netw 2016;19:270-276.
30. Männikkö N. Problematic gaming behaviour in Finnish adolescents and young adults: relation to game genres, gaming motives and self-awareness of problematic use. Behav Med 2017;2:324-338.
31. Nuyens, Filip. Impulsivity in multiplayer online battle arena gamers: Preliminary results on experimental and self-report measures. Journal of behavioral addictions 2016;5:351-356.
32. Helena C, Griffiths MD. Social interactions in massively multiplayer online role-playing gamers. Cyberpsychology & behavior 2007;4:575-583.
33. Dianwen W, Zhilin Y, Zhihua D. Is Sociability or Interactivity more Effective for Enhancing Performance? Findings from a Massively Multiplayer Online Role-Playing Game. Journal of Interactive Marketing 2019;48:106-119.
34. Kou, Yubo, and Xinning Gui. Playing with strangers: understanding temporary teams in league of legends. Proceedings of the first ACM SIGCHI annual symposium on Computer-human interaction in play 2014.
35. Wichstrøm, L, Stenseng F, Belsky J, Soest T, Hygen B. Symptoms of Internet gaming disorder in youth: Predictors and comorbidity. J Abnorm Child Psychol 2019;1:71-83.

Volume: 42, Article ID: e2020016
https://doi.org/10.4178/epih.e2020016

Table 1. General Characteristics of 1,532 First-year Middle School Students in Korea

Variables	Category	n (%) or mean±SD
Gender	Boys	1,036(67.6)
	Girls	496(32.4)
Depressive symptoms		8.3 ±7.2
Aggression[1]		55.3 ±17.0
Average Online-game time (hours/day)	≥2	477(31.1)
	<2	1,055(68.9)
Family type	Non-intact family	138(9.01)
	Intact family	1,394(90.9)
Social economic statue	Low	1,081(70.5)
	High	451(29.5)
Game genres[2]	Role playing game	608(39.7)
	Shooting game	669(43.7)
	Multiplayer online battle arena	599(39.1)
	Simulation game	636(41.5)
	Arcade game	1,001(65.3)
	Sports game	371(24.2)
	Action game	72(4.7)
Risk of Internet gaming disorder	High risk[3]	154(10.1)
	Low risk[4]	1,378(89.9)

[1]Aggression score range : 27-135
[2]Multiple answer available
[3]High risk indicates 10 or higher scores in IGUESS
[4]Low risk indicates less than 10 in IGUESS

https://doi.org/10.4178/epih.e2020016

Table 2. Experience of game genre, daily average time spent and number of game genres

Game Genres[1]	Ever user during the past year	n	Average game time (hours/day)		Number of using game genres (%)[2]				
			Median (Q1-Q3)	p value	1	2	3	4	5+
Role playing game	Yes	608	1.5 (0.7-2.7)	<.001	15 (0.9)	139 (9.2)	188 (12.3)	153 (9.9)	113 (7.4)
	No	924	1.0 (0.4-2.0)		325 (21.2)	327 (21.4)	181 (11.9)	76 (4.9)	15 (0.9)
Shooting game	Yes	669	1.6 (0.7-2.7)	<.001	19 (1.2)	151 (9.9)	199 (12.9)	177 (11.7)	123 (8.0)
	No	863	0.9 (0.3-1.9)		321 (20.9)	315 (20.6)	170 (11.1)	52 (3.4)	5 (0.3)
Multiple online battle arena	Yes	599	1.6 (0.7-2.6)	<.001	31 (2.0)	108 (7.1)	187 (12.2)	162 (10.6)	111 (7.3)
	No	933	0.9 (0.3-2.0)		309 (20.2)	358 (23.4)	182 (11.8)	67 (4.3)	17 (1.1)
Simulation game	Yes	636	1.3 (0.5-2.5)	<.001	43 (2.8)	189 (12.3)	181 (11.9)	126 (8.2)	97 (6.3)
	No	896	1.1 (0.4-2.2)		297 (19.4)	277 (18.1)	188 (12.3)	103 (6.7)	31 (2.0)
Arcade game	Yes	1,001	1.1 (0.4-2.3)	.848	199 (12.9)	276 (18.0)	234 (15.4)	172 (11.2)	120 (7.8)
	No	531	1.2 (0.6-2.3)		141 (9.2)	190 (12.4)	135 (8.9)	57 (3.7)	8 (0.5)
Sports game	Yes	371	1.3 (0.5-2.3)	.539	33 (2.2)	58 (3.8)	105 (6.9)	104 (6.8)	71 (4.6)
	No	1,161	1.1 (0.5-2.3)		307 (20.0)	408 (26.6)	264 (17.2)	125 (8.2)	57 (3.7)
Action game	Yes	72	1.6 (0.8-3.1)	<.001	0 (0.0)	11 (0.7)	13 (0.8)	22 (1.4)	26 (1.7)
	No	1,460	1.1 (0.5-2.3)		340 (22.2)	455 (29.8)	356 (23.2)	207 (13.5)	102 (6.7)

[1]Multiple answer available
[2]Numbers and proportions among 1,532 participants.

Volume: 42, Article ID: e2020016
https://doi.org/10.4178/epih.e2020016

Table 3. Association between experience of game genre and risk of Internet gaming disorder (n=1,532)

Game Genres	Category	Model I	Model II	Model III
Role playing game	Yes	1.69(1.21-2.36)	1.63(1.11-2.41)	1.52(1.03-2.26)
	No	1.00	1.00	1.00
Shooting game	Yes	1.58(1.13-2.21)	1.39(0.94-2.06)	1.23(0.82-1.85)
	No	1.00	1.00	1.00
Multiple online battle arena	Yes	1.38(0.98-1.92)	1.69(1.11-2.57)	1.59(1.03-2.45)
	No	1.00	1.00	1.00
Simulation game	Yes	1.65(1.17-2.29)	1.48(1.02-2.15)	1.51(1.03-2.21)
	No	1.00	1.00	1.00
Arcade game	Yes	1.08(0.75-1.53)	1.05(0.69-1.61)	1.01(0.66-1.53)
	No	1.00	1.00	1.00
Sports game	Yes	0.99(0.67-1.45)	1.06(0.68-1.66)	1.13(0.72-1.78)
	No	1.00	1.00	1.00
Action game	Yes	0.96(0.43-2.13)	0.57(0.20-1.64)	0.56(0.19-1.61)
	No	1.00	1.00	1.00

Model II: Adjusted by Gender, family type, socio-economic status, Depressive symptom, and Aggression

Model III: Adjusted by Gender, family type, socio-economic status, Depressive symptom, Aggression, and Online game genres
